# Morel-Lavallee Lesion Initially Diagnosed as Quadriceps Contusion: Ultrasound, MRI, and Importance of Early Intervention

**DOI:** 10.5811/westjem.2015.3.25148

**Published:** 2015-04-09

**Authors:** Nicholas A. Weiss, Jeremiah J. Johnson, Shane B. Anderson

**Affiliations:** * San Antonio Military Medical Center, Fort Sam Houston, Texas; † San Antonio Military Medical Center, Department of Emergency Medicine, Fort Sam Houston, Texas; ‡ San Antonio Military Medical Center, Department of Radiology, Fort Sam Houston, Texas

## Abstract

Morel-Lavallee lesions (MLL) are rare, closed degloving injuries caused by trauma that delivers a shearing force to the soft tissue most commonly of the hip. If not treated in the acute and subacute setting these lesions are often complicated by re-accumulation of fluid, infection, or chronic pain. We present a unique case of a recurrent, massive medial knee/thigh MLL in which proper treatment was delayed due to initial diagnosis of a quadriceps contusion. We describe the ultrasound and magnetic resonance imaging findings of this patient and based on a review of recent literature propose that the initial management should have included early drainage/debridement, which likely could have prevented recurrence and significantly shortened the clinical course.

## INTRODUCTION

Morel-Lavallee lesions (MLL) are rare injuries that occur due to a traumatic shearing force or crush injury acting on the skin surface that causes a separation of the skin and subcutaneous tissue from the underlying fascia. This mechanism of the injury is referred to as internal degloving. The traditional and most common location of these injuries is the lateral hip/greater trochanter. Other frequent areas include the pelvis, thigh, and knee.^1^ The separation of the subcutaneous tissue from the fascia in MLLs causes a disruption of the lymphatics and blood vessels in the affected region. This precipitates the accumulation of fluid in this newly formed potential space. Subsequently, the formation of a hematomas or seromas occur.^2^ The inflammatory reaction that ensues if these injuries are not treated in the acute phase can organize granulation tissue into a fibrous capsule.^1^,^3^ This capsule impedes the absorption of the fluid and is thought to be the cause of recurrent fluid collection even after drainage,^3^ MLLs are often not diagnosed initially. Kottmeir et al. reported that they are missed up to 44% of the time.^4^ Early detection and treatment of MLLs is vital to circumvent complications such as re-accumulation of fluid, infection-related morbidity, and chronic pain.^5^,^6^ Recent studies advocate for early treatment via drainage and possible debridement of acute and subacute lesions.^2^,^7^,^8^

We present a patient with a massive MLL of the medial thigh that was initially diagnosed as a quadriceps contusion, which caused delayed treatment. We discuss the pertinent ultrasound and magnetic resonance imaging (MRI) findings as well as address the importance of early identification and proper management of these lesions.

## CASE

The patient was a 22-year-old active duty male in the Navy who presented to the emergency department (ED) with a massively swollen, bruised, and painful right thigh/knee after falling down the stairs onto his knee 11 days prior. Before seeking treatment at the ED he saw his primary care provider and was treated with decadron and toradol injections. He was also given oral nonsteroidal anti-inflammatory drug (NSAIDS), muscle relaxants, an ACE wrap, and crutches. The patient denied fever and chills. His medical history was significant only for hypertension. On physical exam he had a large fluctuant fluid collection along the medial aspect of his right thigh as well as diffuse ecchymosis of the leg centered over the knee. There was no joint line tenderness and no ligamentous laxity of the knee joint. He had full range of movement at the knee, and he was neurovascularly intact distally. All compartments of the leg were soft. Plain films of right knee, femur, tib/fib, were significant for soft tissue swelling and no osseous abnormality. Bedside ultrasound in the ED showed approximately 500mL of subcutaneous, anechoic fluid near the vastus medialis. He was diagnosed with a quadriceps contusion and managed with NSAIDs, compressive dressing, knee immobilizer, and follow up with orthopedics.

**Figure 1:**
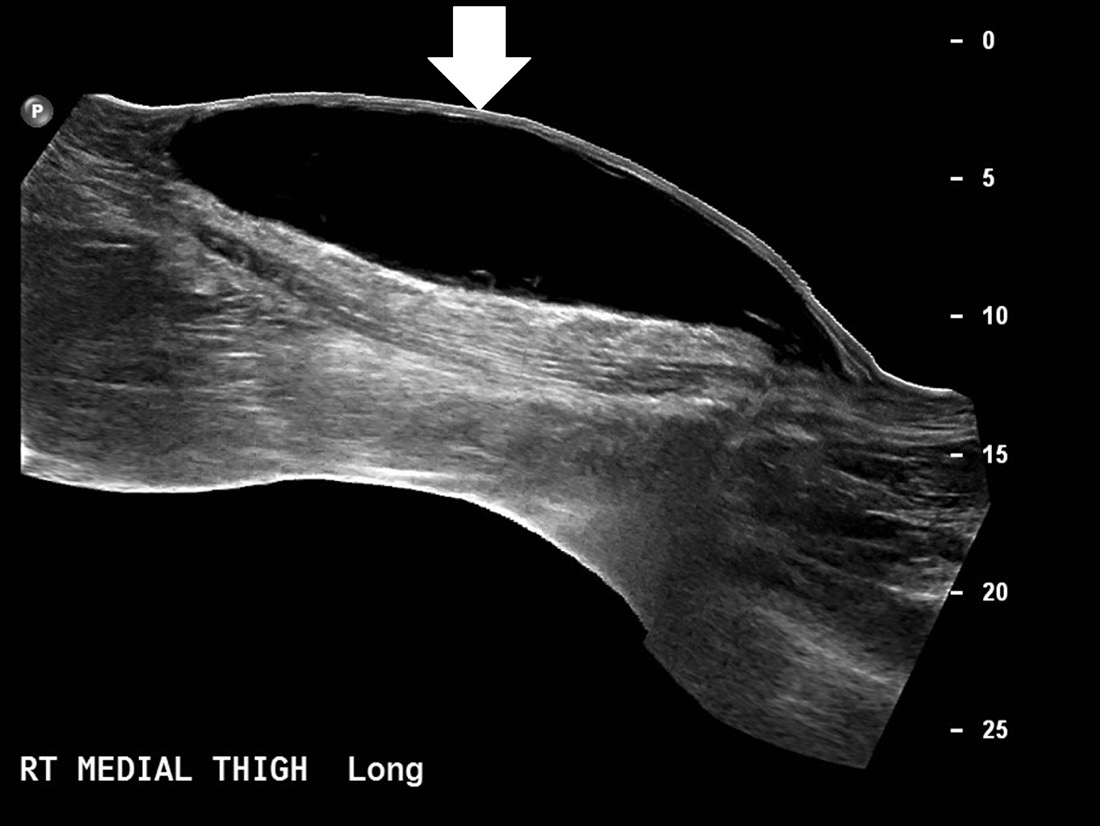
Morel-Lavallee lesion sonography with extended field of view along the long axis of the lesion shows fusiform shape and anechoic texture.

The patient presented again to the ED approximately three weeks later with worsening pain and swelling of the right thigh and knee. Ultrasound demonstrated a fluid collection measuring 26cm cranio-caudid × 6.2cm AP × 13.8cm transverse (Figure 1). The lesion was percutaneously drained and 1900mL of serosanguineous fluid was expressed. Compressive dressing and knee immobilizer were placed and follow up in one week was recommended.

One week later the patient had re-accumulation of the fluid and the decision for surgical irrigation and debridement (I and D) with negative pressure wound dressing placement was made. A pre-operative MRI was obtained (Figure 2a–d). The patient eventually underwent one more surgical I and D with delayed primary closure, and at that time the fluid collection had completely resolved. This was more than a month and a half after his initial presentation to the ED.

## DISCUSSION

This case is clinically significant for two primary reasons. First, the lesion was extremely large for its location. There are very few published accounts of massive MLLs occurring in the medial thigh/knee. To our knowledge our patient’s lesion may in fact be the largest documented in this region, measuring at 26cm cranio-caudal, 6.2cm AP, and 13.8cm transverse and draining 1900cc. Multiple lesions of this magnitude have been described along the lateral thigh/greater trochanter, but after a thorough literature review only two other lesions that possibly were of similar magnitude in the medial thigh/knee were found. Jones et al. presented a large MLL in a 70-year-old women who had been hit by a car. Her lesion measured 30 × 15cm, but the article did not describe a fluid volume.^9^ The other case is of a 26-year- old male who fell on his anteromedial knee during a soccer match. The case simply describes a medial thigh/knee MLL as massive, but does not make comment of measurements.^10^ Most of the lesions caused by trauma to the knee are significantly smaller than the one described on our case. A 2007 study evaluated 27 cases of MLLs of the knee in the National Football Leauge. The largest suprapatellar and midthigh lesions in this study were up to 300mL in size. The mean amount of fluid that could be aspirated from an area of fluctuance in the knee or thigh was only 46mL with a range of 12–120mL.^11^

The second reason this case is clinically significant is that it demonstrates the importance of early identification and proper treatment of MLLs, which are very often not diagnosed initially.^4^ Our patient’s lesion was first thought to be a quadriceps contusion. Complications including recurrence, infection, and chronic pain arise when MLLs are not treated in the acute or subacute window. Although there was evidence present in the history, physical exam, and ultrasound to indicate a MLL, due to its rarity the diagnosis was not made at first. The history detailed a traumatic sheer injury to the soft tissue of the knee. The exam demonstrated diffuse ecchymosis and the hallmark finding of MLLs, a palpable, soft, fluctuant mass over the medial thigh and knee.^10^,^11^ On ultrasound exam the fluid collection was compressible, anechoic and located subcutaneously. In a retrospective study of 21 MLLs of the hip and thigh all demonstrated hypoechoic or anechoic echogencity, were compressible and were located in between the deep subcutaneous fat and the fascia.^5^

Once a fluid collection has been identified as a MLL, research demonstrates that timely intervention via drainage with or without debridement is essential to avoid potential complications. In a 2013 retrospective study of 87 MLLs Nickerson et al. demonstrated that lesions with volumes exceeding 50mL on aspiration were especially prone to reoccur, even after percutaneous aspiration. Specifically, 83% of lesions that drained more than 50ml recurred. This study recommends that lesions with >50ml aspirated require operative drainage via incision and insertion of suction drain.^2^ In a different study of 19 patients with MLLs the authors used operative percutaneous drainage, irrigation and debridement with drain placement to treat large lesions averaging 30×12cm. The study demonstrated prevention of recurrence in all patients treated, and recommended treatment within 3 days if possible.^12^ A similar study used operative percutaneous drainage, debridement, catheter placement and suction of MLL’s at a mean time of 11.9 days from time of injury to intervention. This method was also successful in preventing lesion recurrence in all patients.^13^ Through earlier diagnosis and following the recommendations to operatively drain with or without debridement we propose that our patient may have avoided lesion recurrence and would have healed faster. Although MLLs are admittedly a rare diagnosis, a persistent subcutaneous fluid collection in the setting of trauma should raise clinical suspicion of an underlying MLL.

## Figures and Tables

**Figure 2a:**
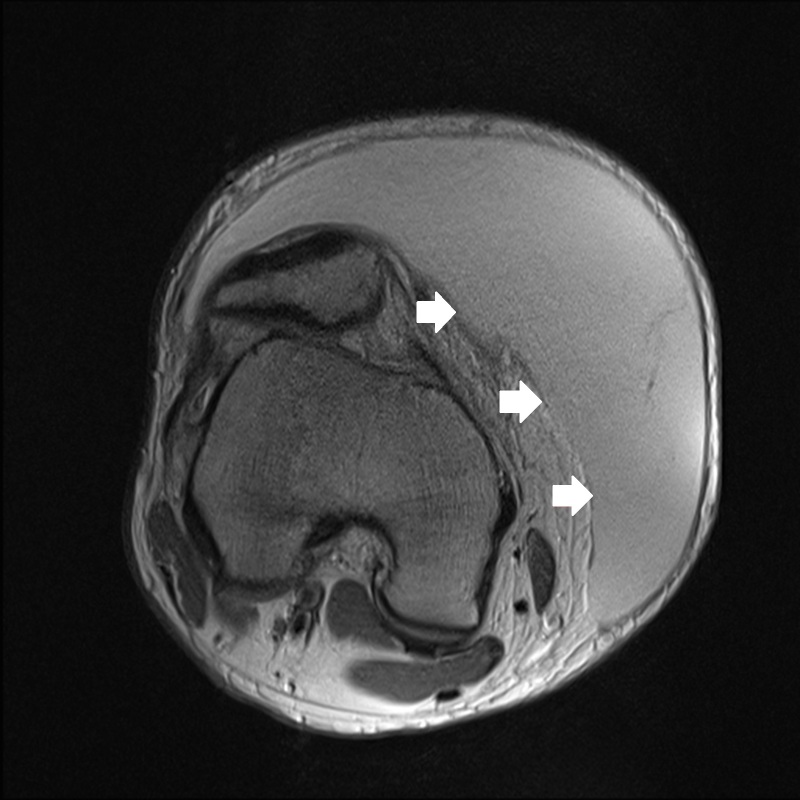
Axial proton density high resolution magnetic resonance imaging shows T2 prolongation in the Morel-Lavallee lesion of the anteromedial right thigh soft tissues.

**Figure 2b:**
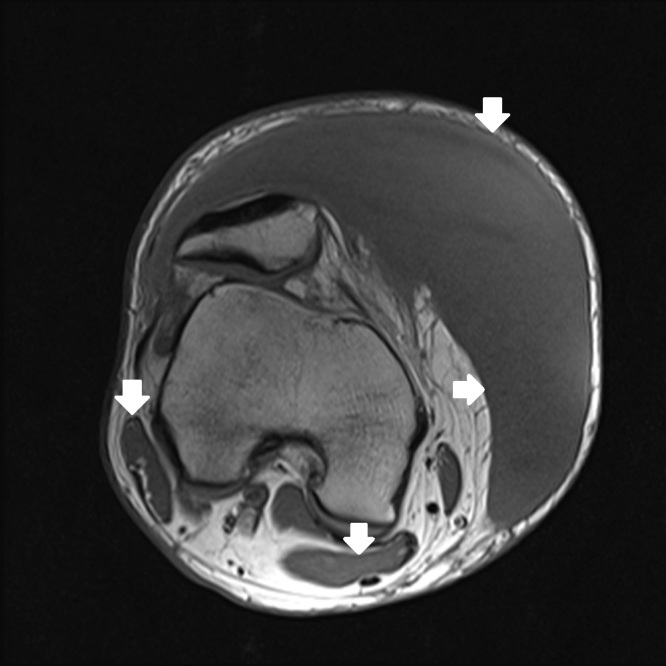
Axial T1-weighted magnetic resonance imaging shows the lesion is isointense to muscle.

**Figure 2c:**
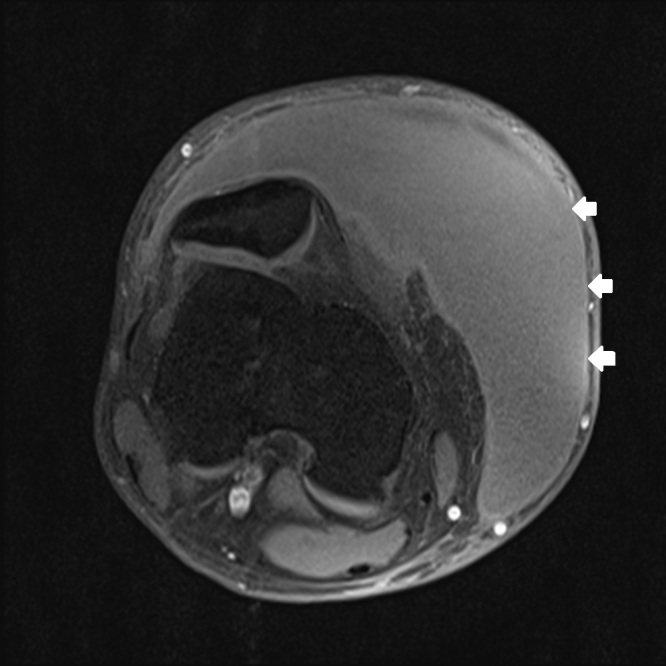
Axial T-1 weighted magnetic resonance imaging with fat saturation shows a capsule of variable thickness (white arrows).

**Figure 2d:**
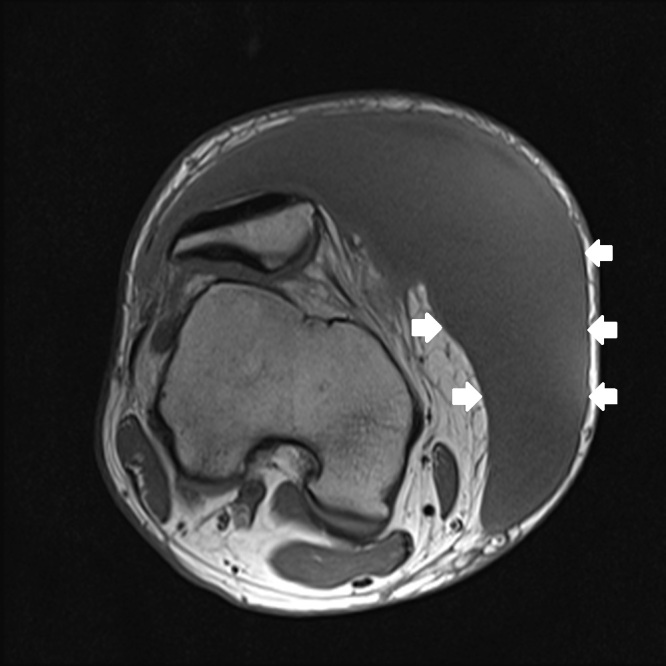
Axial T1-weighted magnetic resonance imaging shows enhancement of the capsule.
